# Barriers and facilitators influencing hearing help-seeking behaviors for adults in a peri-urban community in South Africa: a preventive audiology study

**DOI:** 10.3389/fpubh.2023.1095090

**Published:** 2023-10-17

**Authors:** Thobekile Kutloano Mtimkulu, Katijah Khoza-Shangase, Luisa Petrocchi-Bartal

**Affiliations:** Department of Audiology, School of Human and Community Development, University of the Witwatersrand, Johannesburg, South Africa

**Keywords:** help-seeking, barriers, facilitators, hearing impairment, adults, context

## Abstract

**Objective:**

This study aims to examine the barriers and facilitators to accessing ear and hearing care experienced by adults with hearing impairment in a developing South African context.

**Methods:**

A total of 23 participants were recruited through purposive sampling from an audiology department of a public hospital in peri-urban South Africa. Individual semi-structured interviews were conducted to capture a broad range of perspectives. Data were analyzed through thematic analysis.

**Results:**

Socio-economic factors acted as the primary barrier while structural and health system factors were the main facilitator in influencing participants' journeys toward hearing help-seeking.

**Conclusion:**

Help-seeking decisions made by adults with hearing impairment are impacted by numerous factors. Individual, providers, and environmental factors combine to play a significant role in resolving ear and hearing complaints. Socio-economic and healthcare level gaps reveal the inequalities that affect help-seekers, which, therefore, need to be addressed. The provision of equitable audiology services within hearing health policy is critical for the prevention of severe consequences of hearing impairment. Plans to implement universal healthcare through the National Health Insurance (NHI) by the South African government must include the universal access to preventive audiology services.

## Introduction

The first-ever World Report on Hearing has reported that one in five people live with hearing impairment (1). Prevalence rates have been found to be pervasive in countries with the lowest level of healthcare access and quality, with the African region featuring prominently (2). Global health studies have reasoned that the number of people with hearing loss in low- and middle-income countries (LMICs) is not congruent with the availability of services and resources, resulting in an increase in the years lived with a disability (2, 3).

Disabling hearing impairment has been described as a hearing loss >35 decibels (dB) in the better ear (3). The impact of untreated hearing impairment has adverse consequences on the individual and the society (3, 4). Individuals with hearing difficulties have challenges in communication leading to psycho-social issues and participation restrictions in daily activities (4). At a societal level, higher unemployment rates have been reported due to challenges in obtaining or keeping a job (5). Nevertheless, despite these direct and indirect effects of hearing impairment, those affected still delay in seeking help (6).

The decision-making in help-seeking whether toward or away from resolving hearing difficulties has been described as an iterative process with a “push–pull” effect [(7); p. 198]. In an in-depth Canadian exploratory study, individuals with hearing difficulties were found to have barriers and facilitators in taking a step toward resolving their symptoms (7). During self-assessment, emotional, cognitive, or social factors occur to hinder or influence the hearing help-seeking process. Other hearing help-seeking research studies categorize these elements into audiological and non-audiological factors. Specifically, audiological factors include self-reported hearing difficulties, activity limitations, and participation restrictions; whereas, non-audiological factors include personal elements such as age, gender, attitudes of others, and stigma (6–10). These are the main factors currently described as influencing participants in their journey. However, this evidence emanates from limited publications on this process, where the research papers comprise studies utilizing a combination of face-to-face interviews and literature reviews.

In addition to the dearth of evidence on this subject, further analysis of available studies also reveals that researchers tend to focus on one factor, such as psychological factors, or social support (11–13). For example, Meyer and Hickson (13) identified self-perceived hearing loss as the key determinant for facilitating help-seeking. Meanwhile, a few studies have argued that stigma is the main barrier to resolving hearing difficulties (9, 10, 14). Investigations on the influence of the environment have been limited to social support only (6, 12, 14, 15). This excludes other important factors that may have an additional and equally important impact on the behaviors of individuals with hearing impairment. This indicates that the importance and influence of the context have not been broadly investigated (13, 16). Evidence to support this in hearing healthcare is conspicuous by the limited research outputs.

Another reason for the limited contextual findings is that current publications have been conducted mostly in high-income countries (HICs) with unique healthcare systems as well as better access in terms of resources and professional human resources (ear and hearing care workforce). Ear and hearing care specialists are easily accessible and available, hence self-reported hearing difficulties and psycho-social factors are the strongest determinant influencing help-seeking (8, 12, 13). In addition to a focus on investigating age-related hearing loss, the sample of participants is mostly homogenous in terms of socio-economic position and socio-linguistic and cultural factors (6, 7, 9, 12, 13, 15, 17). The contemporary evidence from these contexts is not applicable to other social contexts. A host of other contextual factors exist from which we can understand the lived experiences of individuals with a hearing impairment.

A few studies on other chronic conditions such as depression and human immunodeficiency virus/acquired immunodeficiency syndrome (HIV/AIDS) have reported on contextual factors acting as either barriers or facilitators (18–20). Results indicate the influence of socio-economic conditions, the severity of illness, access to quality healthcare, and the presence of a comorbid illness, to name a few, as facilitators in the journey to seeking help. Some structural factors such as distance, transport costs, and waiting times were also reported as barriers to help-seeking. This reveals that there are contextually relevant factors; however, the studies mentioned above are based on chronic diseases that present themselves differently from hearing impairment.

South Africa is a diverse country with 11 official languages impacted by a chronic quadruple burden of disease (21), and significant socio-economic inequalities. Large disparities in the provision and access to healthcare coupled with an overburdened public healthcare system that is not able to meet the demands of over 70% of its population means health outcomes will not be favorable for all (22). Similarly, inequalities in socio-economic status exist between population groups, provinces, and socio-economic groupings (23). These social determinants of health and the unequal distribution of resources indicate a high deprivation index (24, 25). However, there may also be areas with a low deprivation index within South Africa as an uneven allocation of resources has been reported across provinces (21), and across the private vs. public healthcare systems that co-exist. In South Africa, all these aspects have been found to determine access to and use of healthcare services (26).

All these contextual factors when considered together contribute to how individuals manage their healthcare condition. The complexity of help-seeking as well as the individual, illness, family, and social context influence decisions toward resolving hearing difficulties (27, 28); thus the importance of the current study aimed at understanding the barriers and facilitators to ear and hearing care help-seeking within the South African context.

## Methods

### Study design

To investigate the experiences and perspectives of adults with hearing impairment, a descriptive qualitative research design was employed. The qualitative research method provided a better understanding of the barriers and facilitators in the context, while the descriptive nature yielded a detailed summary of the experiences by staying close to the data through the words used by the participants (29).

### Participant recruitment

Participants were recruited from an audiology department of a public hospital in Potchefstroom, South Africa. Potchefstroom is a steadily growing peri-urban community within a university town consisting of a mixed and multi-lingual group of inhabitants (30). The departmental appointment book was used as a reference for identifying potential participants according to a set of inclusion and exclusion criteria (31). Specifically, participants had to be 18 years and above, from all genders, and first audiology consultation presenting with a hearing loss of any type, degree, or severity. Participants younger than 18 years of age and those with cognitive and linguistic challenges that impair their ability to consent and participate in the study were excluded. It is also recommended for qualitative health research that clinical settings be used to recruit participants (29), hence the approach adopted in the current study.

To ensure access to different perspectives and a broad range of views, maximum variation sampling was used to select participants (32). Variations that were considered included age, gender, income level, occupation status, cultural beliefs, variety of referral sources, degree of hearing impairment, and experiences with seeking help. These variations were important so that responses to the interview questions in relation to the subject matter captured the individual's experiences as opposed to a homogenous group. This would allow for a comprehensive description of barriers and facilitators according to each participant and therefore reduce a limited range of views. On the day of the assessment, participants were approached, and the research aims were discussed with the invitation to be part of the study. Subsequently, all interested participants gave written voluntary informed consent.

### Data collection

Following a pilot study and guided by data saturation, semi-structured interviews were conducted with 23 participants individually using an open-ended interview guide. Individual interviews were ideal as they allowed the researcher to delve deeper into the personal and social matters related to the subject being studied which may not have been possible in group interviews (31, 33). Although challenges in memory recall and evoking the wrong response were possible and acknowledged, the interviews nevertheless provided powerful insights into participants' lived experiences (31, 34, 35).

All face-to-face interviews were audio recorded before or after the audiology assessment with the participants' consent. Telephonic interviews were also conducted for those participants who were missed during the data collection period, but only where the degree of hearing loss was not a barrier to telephonic communication. Due to the COVID-19 pandemic at the time of data collection (April–June 2021), all precautions were adhered to according to stipulated regulations (36, 37). During this period, the National Institute for Communicable Diseases (NICD, 2021) reported that there was a total number of 1,954,466 laboratory-confirmed cases of COVID-19, with a 26.2% positivity rate, and a number of total fatalities at 60,264 (38).

### Data analysis

Following a review of the current existing literature on the topic, deductive analysis was followed to derive categories for barriers and facilitators. The interviews were transcribed verbatim and analyzed thematically using a deductive approach, ensuring that relevant data were included and irrelevant data were excluded (39, 40). Multiple readings of the raw data were conducted to familiarize oneself with the data and to know the depth and breadth of the content before it could be broken down (39, 41). Following this process, the data were broken down into smaller meaning units and labeled into codes. Coding identified topics, issues, similarities, and differences from the participants' narratives that enabled an understanding of their experiences in terms of barriers and facilitators (39, 42). Similar codes derived from the data were then collated and condensed to develop categories based on previous knowledge as the researcher was re-testing existing data in a new context (43). It was important that these categories correspond with identified categories in the literature to be consistent with this type of analysis and to broaden an understanding of this phenomenon (44). Similarly, when new categories emerged, this contributed to the knowledge of the contextual realities of hearing help-seeking. These categories or themes formed the basis of the results and findings of the study.

Quality control was achieved by applying a recursive method and frequently reviewing the data during analysis. To increase the accuracy of the data, one independent person was used to transcribe 25% of the data (32). The data were also coded independently to ensure the truthfulness of the findings (42, 44). Any differences were openly discussed until a consensus was reached. To increase the credibility of the data, verbatim quotes from the interviews were used to give evidence to the transcription in relation to the aim of the study (32).

As part of the data analysis, codes were also quantified to illustrate the magnitude of the participants' experiences (38). For the quantitative data, measures of central tendency were used to calculate the socio-demographic information (31).

### Ethical considerations

The study conformed to ethical principles laid down in the World Medical Association Declaration of Helsinki (45), with ethics approval having been secured from the University's Human Research Ethics Committee (ME201003) (46).

## Results

### Demographic information

A total of 23 hearing-impaired adult participants, with an average age of 67.8 years (SD ± 15.6) from a fairly equal gender distribution (male=52%; female=48%) and coming from mainly three ethnic groups (Tswana = 57%, Afrikaner = 39%, English = 4%), comprised the demographic profile of the study. The hearing impairment in the sample was characterized by a pure tone average of 49.6 dBHL (SD = 9.1) in the better ear and 56.1 dBHL (SD = 8) in the worse ear—a moderate impairment (3). Audiometric results indicated the degree of hearing impairment ranged from 23.75 dBHL to 91.25 dBHL in the better ear, calculated as a four-frequency pure tone average (4 FPTA). As the South African government considers old age from 60 years, and therefore the sample size being mostly older adults (73.9%*)*, with the average age being 67.8 years, the audiological profile was also considered in relation to age as a causal factor; however, only six participants had a typical age-related hearing impairment as depicted in literature (47, 48). A large majority (91%) of the sample was unemployed. However, this high unemployment number is not surprising considering the age of the sample was mostly economically inactive participants due to them being of retirement age. Nevertheless, eight participants (30.4%) were in the economically active stage of being employable but only two were employed at the time of data collection. None of the participants were self-employed or were students.

### Barriers

#### Socio-economic factors

**Figure 1:**
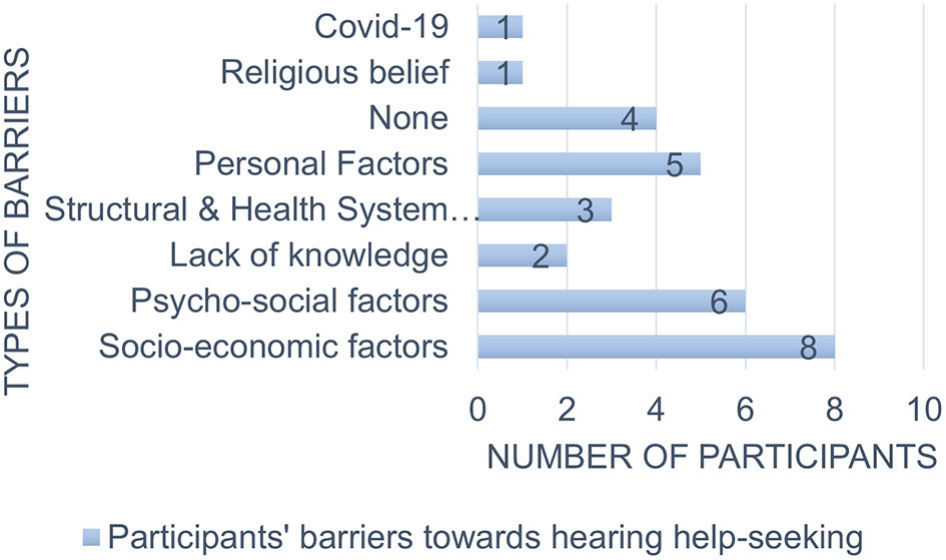
Participants' barriers toward hearing help-seeking (South Africa, 2020-2022).

Figure 1 illustrates the barriers to hearing help-seeking behavior found in the current study.

Among all the participants, the greatest barrier was socio-economic factors. A lack of finances and health insurance was a great limiting factor in accessing help.

Participant 11's wife stated:

“*The only thing was finance! (Pauses). It was just the money.” (Participant 11)*

Participant 4 said:

“*...because you know it's very expensive…and that's why we (pauses), idle because the medical aid is not helping and she's a pensioner. You know (pauses), that's why.” (Participant 4)*

Interestingly, participants were referring to the cost of private audiology healthcare, which is self-funded in South Africa, as opposed to public healthcare which is funded by the government and is free of charge for pregnant women, children under 6, and pensioners. Participants seem to have accessed private audiology care as a last alternate resort. This barrier was considerably more notable for older adults as they aged and with no possibility of employment to pay for these services. This theme raises implications about access in the public healthcare sector, and what challenges exist there to drive these patients to services where they have to pay.

#### Psycho-social factors

Psycho-social factors were the second most commonly reported barrier (26%) to hearing help-seeking. Attitudes toward hearing impairment, healthcare workers, as well as hearing aids contributed to the delay in the participants' journey. From the transcribed interviews, despite the effect of the hearing impairment on communication, participants minimized or denied their hearing difficulties reporting the following:

“*She stopped me herself. She was stubborn. It was hard to admit the hearing loss for her.” (Participant 19) Participant 19 denied the problem while her daughter had acknowledged it*.

While Participant 2 and her daughter minimized the hearing impairment:

“*I think just like me…she took it lightly.” (Participant 2)*

Stigma featured majorly as a psycho-social factor. Denial is also an important consideration in this theme, as accepting conditions that, particularly in this sample, may indicate aging is not easy. Not only did participants have a negative attitude toward their hearing difficulties but the same was observed toward hearing aids. The focus on the appearance and size of the hearing aids, rather than the benefits they might have provided, synergizes well with stigma and negative attitudes.

Participant 9 said,

“*…my brother also had one, but it was also big, and I said I don't want those things, you know, (pauses)… it, it's not me because I, (hesitates), I can't see myself in those.” (Participant 9)*

A negative attitude toward healthcare workers was also observed to be a stumbling block in help-seeking for hearing difficulties. It is important to note that in the South African context, healthcare is accessed not only from Western doctors, but approximately 80% of the South African population consults traditional healers before accessing Western healthcare (49), thus this theme raises an important implication about models of healthcare adopted in this context and how these must be trusted by the populations they serve. Participant 6 reported the following:

“*Ya, you know I'm not a person who likes to go to the doctor. So, I don't trust them actually unless I know that doctor very well.” (Participant 6)*

#### Personal factors

In total, five participants (21.7%) described family responsibilities to dynamics related to age as a barrier to seeking help sooner. Participants were required to prioritize the needs of others before their own. This resulted in their own needs not being met timeously. This is the *Ubuntu* (humanity) principle that is typically the Afrocentric stance toward lifestyle, framed around the Batho Pele (people first) (50).

Participant 3 said:

“*My only prevention was (pauses) my mother. She was in and out…going to and from (referring to hospital admission due to ill health) with her and her situation became*

*(hesitates)” (Participant 3*)

Participant 21 reflected on her decision-making:

“*When you are young, you play with your time. You must immediately seek help.” (Participant 21)*

It is interesting that, in this context, being young and seeking help early for effective intervention and positive outcomes is not viewed as it is in Western contexts, where early intervention is key (51).

#### Structural and health system factors

Interestingly, healthcare workers were highlighted by participants (13%) as a barrier to accessing help. Participants reported a lack of information and care related to their presenting difficulties.

Participant 18, who had sought help from an ENT specialist at the hospital, reported:

“*The doctors do not give you the full information. The sick person tells the doctor, but the doctor must also help.” (Participant 18)*

This finding might also be linked to the well-documented language barrier challenges that exist in South African healthcare, particularly in ear and hearing care where a large majority of the healthcare providers speak English/Afrikaans, languages that are not the first language of the majority of South Africans accessing healthcare (50).

#### No barriers at all

An interesting aspect is that a few participants (17.3%) reported that they did not experience any barriers in their help-seeking journey. From the onset of their journeys to reaching the ear and hearing care providers through the healthcare system did not have any hindrances. Even when participants had been accompanied by family members, they still identified no barriers to report. One would think that the costs incurred in having to come with a family member, such as additional transport costs, loss of income if they have to take a day's leave, and so on, as barriers; however, in contexts where this has been *normalized* it may not come up as such. This, arguably, may be what is happening in this instance.

Participant 14, who was accompanied by his son, stated:

“*There were no challenges. Everything was easily accessible.” (Participant 14)*

“*I don't recall having any barriers. It's just that…ai, I don't know. I would be lying.” (Participant 15)*

Finally, COVID-19 (3.4%), lack of knowledge (8.7%), and religious beliefs (3.4%) were the least reported barriers.

Participant 8 was about to seek help when the South African government declared a level five lockdown stipulating that everyone must stay home as part of COVID-19 spread prevention measures. A lack of knowledge about ear and hearing prevention and cure prevented participants from seeking help. Only one adult reported that she always believed that God would heal her.

### Facilitators

**Figure 2:**
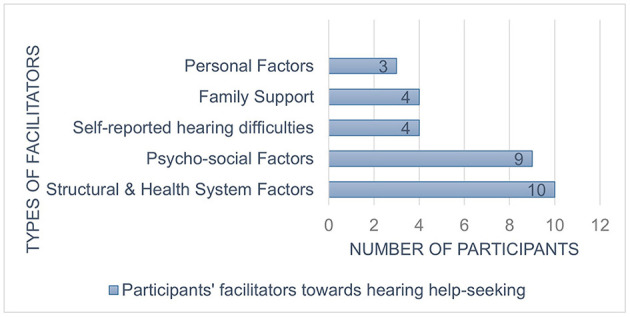
Participants' facilitators toward hearing help-seeking (South Africa, 2020-2022).

Five factors were identified when participants were asked what influenced their journeys, and these are depicted in Figure 2.

#### Structural and health system factors

A majority of participants (43.4%) reported structural and health systems as the main facilitator toward seeking help. The availability and support of healthcare workers, being able to access diverse services under one roof, knowing healthcare workers from the community where the patients come from, and the ease of access to health services were reasons that influenced their help-seeking.

“*The doctors, (pauses) the doctors there. (pauses) They said, you have to look after your hearing, (hesitates) have to look after your hearing because you don't hear what we're saying.” (Participant 1)*

Participant 2, an 89-year-old female participant, accompanied by her daughter stated, addressing the HCW in an African familial term (Ous):

“*I think the help was Ous (HCW name) and to come here to (Hospital name). ‘I think the help was that Ous (HCW name) referring us here.” (Participant 2)*

Participants were able to access the health services they required without being burdened about their affordability.

“*It's wonderful to get to (pauses) to know that you can get help for it without going to pay that kind of money.” “It's so easy to find help.” (Participant 23)*

Even when participants were accessing the hospital services for other reasons, the availability and help of healthcare workers influenced them to pursue interventions despite having already sought help. Similarly, Participant 20, who was still looking for a solution for his hearing difficulties despite his reportedly long journey, stated:

“*To come to (hospital name) is because of I received a pamphlet at the vaccination site. Oh! there is an (pauses) let me try somewhere maybe they can see something different.” (Participant 20)*

#### Psycho-social factors

As reported with barriers, psycho-social factors negatively influenced participants in their journey. Similarly, these factors were the second highest facilitating factor for participants. Positive and negative attitudes toward the hearing impairment, sources of motivation, and the hearing aid itself were described as factors that moved participants forward to resolving their hearing difficulties.

Participant 12's daughter-in-law stated:

“*It is frustrating for him and my mom.” (Participant 12)*

“*…and now the fact they say it's smaller things (referring to the hearing aids), it will be bearable for me. I can grow my hair a little bit longer, things like that, so I can hide it. So, they don't see it.” (Participant 9)*

Participant 22, an 89-year-old male, reported:

“*I simply thought to myself like, the doctor was telling me to go. I simply said to myself, ya, this is a great help for me.” (Participant 22)*

#### Self-reported hearing difficulties

These were described by four participants (17.3%) as the main catalyst for seeking help. Difficulty in communicating with others and constant auditory symptoms affected their quality of life which compelled participants onward in their journeys.

Participant 19 had this to say:

“*It's, talking to somebody and I don't understand what they say or don't hear what they say. That's the main thing for me to say…well I need some help.” (Participant 9)*

Participant 13 complained of activity limitations by reporting that:

“*It's the news, I can't hear well when listening to the TV or when I'm with people. I cannot hear what they are saying.” (Participant 13)*

Finally, *family support* (17.3%) and *personal factors* (13%) positively influenced help-seeking. Participants were either encouraged by their families to seek help or they sought help by themselves. Support was mostly from female members of the family who also accompanied most of the older adults, and this is typically reported in African contexts where caregivers tend to be women in the families and communities (52). Personal factors that were mentioned included age, social support, and a traumatic event. These factors enabled participants to seek and find ear and hearing care helpers.

Participant 4's daughter felt that it was her responsibility as she stated:

“*I made the appointment. I brought her here. We booked her in, and you just helped us now.” (Participant 4)*

Participant 12 was 82 years of age when he decided to seek help. Although he was the only one where age was mentioned in this largely older adult cohort, Participant 12's daughter explained his condition as follows:

“*He's old and sick.” (Participant 12)*

Participant 18, who had consulted an ENT specialist, explained that:

“*An incident helped me to get to the Audiologist. Had it not been for the assault at the taxi rank…I really don't know.” (Participant 18)*

## Discussion

This study aimed to examine the barriers and facilitators to accessing ear and hearing care by adults with hearing difficulties within the context of a middle-income country as part of their help-seeking behaviors. South Africa is a linguistic and culturally diverse country with unique socio-economic disparities, thus findings from the current sample may arguably be generalizable as the demographic profile is a fair representation of adults accessing public ear and hearing healthcare services in South Africa (53, 54).

In examining barriers in participants' contexts, socio-economic factors were found to be the major limiting factor in seeking help for ear and hearing difficulties. The unavailability and limited finances and access to medical insurance also correlated with the reported occupational status of the participants. This finding is consistent with a South African study that reported those with a lower income had less money to spend on healthcare and therefore waited longer to seek help (20). However, it could be argued as to the reasons for this factor as audiology and other healthcare services are charged at minimal or at no cost in the public healthcare sector (55). Public healthcare services in South Africa are overburdened and understaffed with excessive waiting times. This may have influenced participants help-seeking choices toward private healthcare though this came at an unaffordable cost to them (20). In contrast, current research in hearing help-seeking has not reported this finding as authors gathered data from HICs (7–9, 12). Rather, limited evidence from the available research has shown psycho-social factors as the most salient barrier to seeking help in these environments (6, 8–10, 14, 15). Although healthcare is reasonably affordable in the public healthcare sector, and mostly free for pensioners, money for transport to come to the healthcare facility could be a significant barrier as well, particularly because no subsidized public transportation exists in South Africa, and private funding of your own and that of the person accompanying you to the hospital is expensive. The current finding is significant in relation to the importance of research investigating hearing help-seeking behaviors in different contexts.

In the current study, psycho-social factors were the second barrier for participants. It is interesting to note that, in a different context, emotional factors would not be a major influence on individuals despite experiencing the physical and life-changing effects of hearing impairment in a similar fashion. Research in South Africa on non-auditory chronic diseases reported a combination of structural and knowledge factors with psycho-social factors following only the two factors as delaying help-seeking (18, 20). South Africa has large income inequalities and corresponding disparities; therefore, it is possible that psycho-social factors would not be the main factor influencing participants' journeys (56). In multi-cultural contexts, models of healthcare—from traditional healers to Western providers—can have an impact on health behaviors. The reality and consequences of these co-existing models need to be explored and acknowledged in these environments. Nevertheless, this indicates that events around the participant, whether at an individual or societal level, influence the decisions of help-seekers hence the importance of contextual investigations.

From the individual, personal factors contributed to delaying participants on their journeys. A literature review identified personal factors as far as age, gender, and level of education as influencing hearing help-seeking (29). This is in contrast to participants in this study who described personal factors related to family dynamics as a barrier to seeking help. Such dynamics are typical in multi-lingual and multi-cultural contexts as research from other countries has reported on the influence of patriarchal, communal systems, and work commitments in seeking help (56, 57). This difference in personal factors reflects the impact of the cultural context and the community surrounding the individual and therefore must be considered in future research as it has the potential to influence practice.

Interestingly, three participants (13%) reported structural and health system factors as a barrier to seeking help. In relation to being referred to audiology services, health workers' behaviors, abilities, and quality of care fell short in resolving their hearing difficulties according to their expectations as they were insufficiently helped. This is in line with the current status of healthcare in South Africa and the existing socio-economic inequalities (21). A significant challenge in demand vs. supply of health services and a lack of universal healthcare for all citizens makes this barrier not surprising (22). In contrast, a literature review on this subject reported only health system factors related to healthcare professionals stigmatizing hearing impairment (13). When considering the context and living conditions of adults with hearing impairment, this implies that provider factors also contribute to barriers in patients' journeys. Systematic language barriers that impact health-seekers must be addressed for better health outcomes. Furthermore, health systems need to be strengthened with an adequate supply of resources in order to prevent them from delaying and worsening illness (58).

In view of the diverse socio-economic conditions of participants, it is interesting that a few participants (17.3%) reported experiencing no barriers in their help-seeking journey. This is a unique outcome from the study despite the different pathways used to access audiologists. To the best of the researchers' knowledge, there is no evidence to support or contrast this finding. However, this indicates that further contextual investigations are required in hearing help-seeking as there are possibly many unknown barriers in participants' lives.

COVID-19, lack of knowledge, and religious beliefs were the least reported barriers. The response by the global community to preventing the spread of the Coronavirus cannot be underestimated despite restrictions not being placed on healthcare services. One study found that chronic medical conditions influence help-seeking behaviors (18). Though not chronic but serious, the effect of this disease shows that any social condition has the potential to influence the journeys of help-seekers. The researchers also acknowledge the small sample size in influencing this finding. Nonetheless, more studies are required as hearing help-seeking research has not investigated the impact of chronic diseases on help-seeking.

Hearing help-seekers also reported a lack of knowledge of audiology services as a barrier. This is consistent with South African studies that reported participants not knowing where to go for their specific chronic illness (18, 57). Low health literacy has been reported to affect health decision-making. When combined with adverse social determinants of health this can lead to poorer health outcomes (59). Similar findings have not been reported in the current hearing help-seeking research perhaps because ear and hearing specialists are easily accessible and higher rates of health literacy exist in developed contexts (60). Nevertheless, hearing health awareness is required at a primary level, especially in preventive audiology for the whole population in all contexts.

The impact of religious beliefs on a participant's help-seeking journey influenced resolving of hearing difficulties. Patterns of help-seeking related to cultural practices have been reported by research papers on non-auditory chronic diseases (56). Unfortunately, there is a lack of research on the influence of culture from the available hearing help-seeking research studies (16, 61). People emerge from cultural contexts and there is therefore a need to investigate the influence of cultural factors in hearing help-seeking behavior research (61).

Regarding facilitators, structural and health systems and psycho-social factors were the main influences in moving participants forward in their journeys. The care and concern of healthcare workers, their knowledge of audiology services, and the access, availability, and affordability of services promoted help-seeking. This is interesting when considering that health system factors were also barriers for participants in this study. A previous study in the same developing context (Uganda) supports this finding (54). In a country with a poor perception of public healthcare services, limited resources, and a quadruple burden of disease (22, 62), this positive finding provides insight into the role that healthcare workers play in patients' help-seeking behaviors. A sense of agency should drive empathy toward patients' needs in healthcare delivery. Hearing help-seeking research has not reported on health system factors perhaps because available studies have focused on the patient as the decisive agent (29, 63). Hence, psycho-social factors have been stated as playing a prominent role in facilitating help (7, 8, 10, 12, 13). This is in contrast to the current study which ranked this factor second as a barrier and a facilitator. It could be said that social context and healthcare capacity distinctly influence help-seeking (61). Participants were affected emotionally but the help they received in an environment with health system challenges made a bigger difference to their journeys. Future studies must investigate the association between contextual and emotional influences in adults with hearing impairment.

Self-reported hearing difficulties also influenced participants (21.7%) to seek help. Quality of life changes as the hearing loss progressed was a deciding factor to resolve the loss of function. Similar findings have been reported by current hearing help-seeking research (12, 15, 29). However, the fact that self-reported hearing difficulties were not the major facilitator among participants in this current study as it appears in previous studies, supports the view that contextual investigations are required. Though hearing difficulties are experienced the same, help-seeking is not similar across all contexts (64). Clinicians and researchers must consider the environment from which individuals come in order to understand their help-seeking behavior patterns.

A few participants reported on family support and personal factors as facilitators in their help-seeking. These two factors are often seen as one factor in hearing help-seeking research; however, the type of personal factors was different from family support in this current study. Families and acquaintances supported participants by referring, accompanying, or finding audiology services for them. Personal circumstances also helped participants to seek help sooner. Contrasting results have been reported in the current literature on these factors. While some studies found family support and personal factors to be a catalyst to moving help-seekers forward (7, 13, 29), other studies reported these factors to be barriers to help-seeking (6, 10, 14). In addition, Saunders et al. (9) reported family support as one of the nine primary factors negatively influencing help-seeking. Research in South Africa has supported the current study's positive findings (18). Even though the results are from a few participants, the researchers postulate that social factors especially the influence of cultural norms of caregiving strongly influence hearing help-seekers through this iterative process. Hearing health policy needs to also target interventions at a community level to improve health outcomes such as prevention and early interventions for diseases.

This study is not without limitations. Though important contextual findings have been reported, it can be argued that the sample size was small although data saturation was reached. Researchers could have added more than one site for participant recruitment for diversity in contextual realities within the same country. Furthermore, it is acknowledged that recruitment from an audiology department of a public (state funded) hospital has introduced a selection bias toward respondents with possibly greater barriers than those from a private (self and medical aid funded) hospital. Representation, accessibility, turnover, and patient satisfaction are factors that could have been influenced by the current recruitment strategy. The study also consisted mainly of older adults which reduced the heterogeneity of the study. Interview bias could have also influenced the process as the main interviewer is also a healthcare worker.

## Conclusion

This study examined the barriers and facilitators influencing adults with a hearing impairment from a peri-urban community in South Africa. Results revealed that socio-economic factors and structural and health system factors were the main barriers and facilitators, respectively. These findings are insightful and provide valuable evidence of the influence of the contextual environment and individual help-seeking behaviors (65). Help-seeking behavior is inherently complex and future studies need to consider contextual factors on illness behavior, whether local, national, or global. Results from the immediate context will contribute toward a more contextually relevant hearing health practice.

## Data availability statement

The original contributions presented in the study are included in the article/supplementary material, further inquiries can be directed to the corresponding author.

## Ethics statement

The studies involving human participants were reviewed and approved by University of the Witwatersrand Human Research Ethics Committee (Protocol Number: ME201003). The patients/participants provided their written informed consent to participate in this study.

## Author contributions

Conceptualizing, designing the study, data analysis, and writing the first draft: TKM. Supervision: KK-S and LP-B. All authors contributed to interpretation of findings and critical review of the manuscript, read, and approved the final manuscript.
